# Impact of the introduction of chikungunya and zika viruses on the incidence of dengue in endemic zones of Mexico

**DOI:** 10.1371/journal.pntd.0009922

**Published:** 2021-12-02

**Authors:** Larissa Fernandes-Matano, Irma Eloisa Monroy-Muñoz, Hector Daniel Pardavé-Alejandre, Luis Antonio Uribe-Noguez, María de los Angeles Hernández-Cueto, Teresita Rojas-Mendoza, Clara Esperanza Santacruz-Tinoco, Concepción Grajales-Muñiz, José Esteban Muñoz-Medina

**Affiliations:** 1 Laboratorio Central de Epidemiología, Instituto Mexicano del Seguro Social, Mexico City, Mexico; 2 Escuela Nacional de Ciencias Biológicas, Instituto Politécnico Nacional, Mexico City, Mexico; 3 Laboratorio de Genómica, Departamento de Genética y Genómica Humana, Instituto Nacional de Perinatología “Isidro Espinosa de los Reyes”, Mexico City, Mexico; 4 Coordinación de Control Técnico de Insumos, Instituto Mexicano del Seguro Social, Mexico City, Mexico; 5 División de Laboratorios de Vigilancia e Investigación Epidemiológica, Mexico City, Mexico; Oregon Health and Science University, UNITED STATES

## Abstract

**Background:**

With the arrival of chikungunya (CHIKV) and zika (ZIKV) viruses in Mexico, there was a decrease in diagnosed dengue virus (DENV) cases. During the first years of cocirculation (2015–2017), the algorithms established by epidemiological surveillance systems and the installed capacity limited us to one diagnostic test per sample, so there was an underestimation of cases until September 2017, when a multiplex algorithm was implemented. Therefore, the objective of this study was determine the impact of the introduction of CHIKV and ZIKV on the incidence of diagnosed DENV in endemic areas of Mexico, when performing the rediagnosis, using the multiplex algorithm, in samples from the first three years of co-circulation of these arboviruses.

**Methodology and principal findings:**

For this, 1038 samples received by the Central Laboratory of Epidemiology between 2015 and 2017 were selected for this work. Viruses were identified by multiplex RT-qPCR, and the χ^2^ test was used to compare categorical variables. With the new multiplex algorithm, we identified 2.4 times the rate of arbovirosis as originally reported, evidencing an underestimation of the incidence of the three viruses. Even so, significantly less dengue was observed than in previous years. The high incidence rates of chikungunya and Zika coincided with periods of dengue decline. The endemic channel showed that the cases caused by DENV rose again after the circulation of CHIKV and ZIKV decreased. In addition, 23 cases of coinfection were identified, with combinations between all viruses.

**Conclusions and significance:**

The results obtained in this study show for the first time the real impact on the detected incidence of dengue after the introduction of CHIKV and ZIKV in Mexico, the degree of underestimation of these arboviruses in the country, as well as the co-infections between these viruses, whose importance clinical and epidemiological are still unknown.

## Author summary

Mexico is considered an endemic country for DENV as the number of cases each year is high. With the arrival of the CHIKV and ZIKV, this number decreased considerably. This led us to suspect that this decrease was not coincidental and could be attributed to two factors: the lack of expertise on the part of health teams to identify the clinical etiological agent and/or other complex ecological factors. Therefore, we decided to reanalyze 1038 suspected cases of dengue, chikungunya and Zika samples using a test that could identify any of the three viruses in a single reaction. We discovered that there was indeed an underestimation of cases, mostly of chikungunya and Zika but, contrary to what we imagined, a lower degree of dengue. We were also able to detect cases of coinfection, which had never been reported in Mexico. Thus, the present study lays the foundations for a better preparation for the arrival of other arboviruses, showing the need for the development and implementation of multiplex diagnostic techniques, in addition to providing more precise epidemiological information on this historical moment of co-circulation of these three arboviruses in our country.

## Introduction

Arboviruses (for arthropod-borne viruses) are a heterogeneous group of viruses that, as a common characteristic, require the participation of arthropod vectors for their transmission between hosts [1]. Among the most important arboviruses in recent years are dengue virus (DENV), chikungunya virus (CHIKV), and zika virus (ZIKV) [2]. The four serotypes of DENV that are currently known (DENV 1–4) and ZIKV are flaviviruses belonging to the *Flaviviridae* family, while CHIKV is an alphavirus belonging to the *Togaviridae* family [3,4]. The clinical manifestations caused by these viruses are not specific and often resemble each other [5]. This similarity makes it difficult to make a diagnosis based only on the signs and symptoms.

In Mexico, before the introduction of CHIKV and ZIKV (2014 and 2015, respectively), the diagnostic algorithm used by the National Network of Public Health Laboratories focused solely on the detection of DENV [6]. From 2015 through the third quarter of 2017, the molecular diagnostic algorithm underwent several changes to enable it to detect new viruses [7,8]. Two new uniplex RT-qPCR techniques were added, one for CHIKV in 2015 and one for ZIKV in 2016. Finally, in the fourth quarter of 2017, all these techniques in uniplex format were replaced by one in multiplex format, which detects DENV, CHIKV, and ZIKV simultaneously [9].

Before the multiplex algorithm was implemented, there was a restriction that the physician could only choose one of the uniplex techniques, based on the clinical manifestations of the patient. This caused an underestimation in the number of cases of arbovirosis, which in the case of dengue was detected both in the LCE, and in the results issued by the General Directorate of Epidemiology, which show that from 2015 to 2017, the number of total cases of dengue was significantly lower than in the three years before the arrival of CHIKV and ZIKV (2012–2014), decreasing from an average of more than 88 thousand cases per year to slightly more than 50 thousand per year during 2015–2017 [10–15].

The lack of knowledge about the true degree of underestimation due to the diagnostic algorithm made it difficult to establish the true extent of these 3 arboviruses, the true impact on the number of dengue cases and to know the possible existence of coinfections, which could have important implications for the epidemiology and evolution of these viruses. For this reason, in this study, we set out to perform the rediagnosis using the multiplex algorithm in samples of the first 3 years of co-circulation of DENV, CHIKV and ZIKV and thus try to solve these important epidemiological questions.

## Methods

### Ethics statement

Human serum specimens were an excess of samples collected during routine passive surveil- lance activities of the Central Laboratory for Epidemiology (LCE, “Laboratorio Central de Epidemiologia”), Instituto Mexicano del Seguro Social in Mexico City. All specimens were de- linked from any personal identifiers prior to the commencement of the study.

The Study was approved by the Ethics and the Research Committees of the National Com- mittee of Scientific Research of the Instituto Mexicano del Seguro Social with the registration number R-2018-785-013

### Study design

A cross-sectional study was proposed, which was conducted on 1038 serum samples (346 samples from each year: 2015, 2016, and 2017) that were selected from a simple random sampling of the biobank of LCE. The samples came from three states of the Mexican Republic considered endemic for dengue (Oaxaca, Quintana Roo, and Veracruz) and were sent to LCE to confirm the diagnosis of dengue, chikungunya, or zika. All selected samples complied with one of the operational definitions of a probable case of infection by DENV, CHIKV, or ZIKV, established in the Guidelines for the Laboratory Surveillance of dengue and Other Arbovirus Diseases (2017).

### Determining the presence of viruses by RT-qPCR in the selected samples

Total nucleic acids were obtained from 200 μL of serum using the automated MagNa Pure LC 2.0 instrument (Roche, Rotkreuz, Switzerland) and the MagNa Pure LC Total Nucleic Acid Isolation Kit (Roche, Rotkreuz, Switzerland), with which total nucleic acids (RNA and DNA) were obtained according to the manufacturer’s instructions. The viruses were identified in the selected samples with the TaqMan Zika Virus Triplex Kit (Thermo Fisher Scientific, Massachusetts, USA), which detects DENV, CHIKV, and ZIKV in a single reaction. Cyclophilin A (*PPIA*) was used as the internal control. Due to the properties of the kit, only 25 μL of RNA sample was directly added to the lyophilized kit. The amplification reaction was performed in a 7500 Fast Real-Time PCR System (Applied Biosystems, California, USA) under the following thermocycling conditions: one cycle of 50°C for 20 min, one cycle of 95°C for 2 min, and 40 cycles of 95°C for 15 s and 60°C for 1 min.

### Serotyping of DENV

The SuperScript III Platinum One-step RT-qPCR System kit (Invitrogen, California, USA) was used in the 7500 Fast Real-Time PCR System. Specific primers and probes were used for each of four DENV serotypes, and the primers and probe of the human RNase P (RP) gene used as an internal control (S1 Table). The serotypes were evaluated in a multiplex reaction with the following reaction mixture: 12.5 μL of 2x Reaction Mix, 0.25 μL of each of the primers D1 and D3, 0.125 μL of each of the primers D2 and D4, 0.045 μL of each probe, 0.5 μL of enzyme, 5.32 μL of RNase-free water, and 5 μL of RNA. The thermocycling conditions were 1 cycle of 50°C for 30 min, followed by 1 cycle of 95°C for 10 min, 45 cycles of 95°C for 15 s and 60°C for 1 min [16].

### Reaction and interpretation controls

RNA lyophilizates (AmpliRun, Vircell, Granada, Spain) were used as positive controls for all viruses evaluated. A positive result was accepted when any sample presented amplification for any of the viral markers (Ct <38) plus the internal control or RP control, and a negative was when a sample had no amplification of the viral markers but did have amplification of the internal control or RP control.

### Confirmation of coinfection cases

For samples in which the presence of more than one virus was detected, confirmation was carried out by capillary sequencing. To generate the fragments to be sequenced by RT-PCR, specific primers were designed for each of the viruses, as shown in Table 1, and the AgPath-ID One-Step RT-PCR Reagents kit (Applied Biosystems, California, USA) was used in a Veriti Thermal Cycler (Applied Biosystems, California, USA). The reaction mixture used was 12.5 μL of 2x Reaction Mix, 4.5 μL of RNase-free water, 1 μL of each of the primers, 1 μL of enzyme, and 5 μL of RNA. A total of five different amplification conditions were used: For reverse transcription and polymerase activation, in all cases, one cycle of 45°C for 60 min and one cycle of 95°C for 10 min were used. After that came 40 cycles of 95°C for 30 s and 55.8°C for 30 s (DENV 1 and DENV 4); 55.9°C for 30 s and 60°C for 70 s (DENV 2); 52.5°C for 30 s and 60°C for 80 s (DENV 3); 59°C for 30 s and 60°C for 1 min (CHIKV); or 52.3°C for 30 s and 60°C for 80 s (ZIKV). These variable cycles were followed by one cycle of 60°C for 1 min. Last was an additional cycle of 60°C for 1 min in all cases.

**Table 1:** Sequences and working concentrations of primers designed for the confirmation of coinfections.

Virus to Identify	Primer	Sequence (5′-3′)	Concentration (μM)
**DENV 1**	FP	GGG GCG ACA GAA ATC CAG AC	10
**DENV 1**	RP	GCC TGG AAT TTG TAT TGC TCT GTC	10
**DENV 2**	FP	ATG GTA GAC AGA GGA TGG GGA AAT	10
**DENV 2**	RP	GTT CTG CTT CTA TGT TGA CTG GGC	10
**DENV 3**	FP	AGG AGC AGG ACC AGA ACT ACG	10
**DENV 3**	RP	GCC TCG AAC ATC TTC CCA AT	10
**DENV 4**	FP	TGC CGG ACT ATG GAG AAC TAA CA	10
**DENV 4**	RP	TCA CTA TGT AGC TGT CCC CAA AGG	10
**CHIKV**	FP	CAT CAG CAT ACA GGG CTC ATA CC	10
**CHIKV**	RP	GCT GCA CAG TGT ACT TGT GTA GAA CA	10
**ZIKV**	FP	GTG GGG AAA AAA GAG GCT ATG	10
**ZIKV**	RP	CAT ATT GAG TGT CTG ATT GCT TGT C	10

PF: forward primer; PR: reverse primer.

Purification was performed using ExoSAP-IT (USB Corporation, Ohio, USA). The sequence reaction was carried out using the BigDye v3.1 Terminator Sequencing Cycle Kit (Applied Biosystems, Cheshire, England). Each fragment was sequenced with both primers, forward and reverse, at a concentration of 3.2 μM. The sequence reactions were purified using the Dye Ex 2.0 Spin kit (Qiagen, W. Sussex, England) and run in the ABI 3130 Capillaries sequencer.

### Estimation of the number of cases and incidence rate in the three Mexican states

As a suggestion of the number of cases that may have existed in the states studied, the incidence rate per 100,000 inhabitants / year was calculated from the analyzed samples (S1 Fig). The proportion of positive cases in the sample was taken into account and was related to the number of suspected cases notified and sent to the LCE during the study seasons, as well as the number of inhabitants in each state in each year.

### Generation of the endemic channel

The endemic channel was generated by the quartile’s method, which is used in the Division of Epidemiological Surveillance Laboratories of the Mexican Institute of Social Security. The estimated cases of dengue were ordered by months, corresponding to the years 2015–2017 (years of co-circulation of DENV, CHIKV and ZIKV), calculated as explained in the previous subsection. From the data set for each month, quartiles 1 (25th percentile; success zone), 2 (50th percentile; safe zone) and 3 (75th percentile; alarm zone) were calculated. With the obtained values, a stacked line graph was generated with the Microsoft Excel 2010. Then, the 2018 estimated dengue data, generated at the Central Laboratory of Epidemiology, were plotted on the previously generated stacked line plot.

### Statistical analysis

Descriptive statistics were used to analyze the incidence of the viruses included in the study. The χ^2^ test for homogeneity, independence, and goodness of fit and Fisher’s exact test were used to compare categorical variables (p<0.05 was taken as significant). The analyses were performed with IBM SPSS Statistics 24.0, and the graphs were generated with Microsoft Excel 2010.

## Results

### Demographic analysis

Of the 1038 samples analyzed, 398 were from male patients (38.3%) and 640 were from females (61.7%). The mean age was 32.6 years, ranging from 0 to 86 years. The population was divided into four age groups according to the health records of the Mexican Social Security Institute (IMSS). Following this classification, the samples were distributed as follows: 99 correspond to children aged 0 to 9 years (9.5%), 106 to young people aged 10 to 19 years (10.2%), 743 to adults aged 20 to 59 years (71.6%), and 90 for adults aged 60 years or older (8.7%). The analyzed samples were randomly collected from three Mexican states considered endemic: Oaxaca (12.9%), Quintana Roo (8.6%), and Veracruz (78.5%) (Table 2).

**Table 2:** Demographic data of the samples included in the study.

	Total N = 1038 n (%)	2015 N = 346 n (%)	2016 N = 346 n (%)	2017 N = 346 n (%)
**Sex**				
Male	398 (38.3)	137 (39.6)	110 (31.8)	151 (43.6)
Female	640 (61.7)	209 (60.4)	236 (68.2)	195 (56.4)
**Age group**				
0–9 years	99 (9.5)	28 (8.1)	22 (6.3)	49 (14.2)
10–19 years	106 (10.2)	28 (8.1)	38 (11.0)	40 (11.5)
20–59 years	743 (71.6)	251 (72.5)	265 (76.6)	227 (65.6)
≥60 years	90 (8.7)	39 (11.3)	21 (6.1)	30 (8.7)
**State**				
Oaxaca	134 (12.9)	40 (11.6)	39 (11.3)	55 (15.9)
Quintana Roo	89 (8.6)	48 (13.9)	33 (9.5)	8 (2.3)
Veracruz	815 (78.5)	258 (74.5)	274 (79.2)	283 (81.8)

N: total samples analyzed; n: samples identified.

### Positivity

The positivity analyses were performed per year, and the percentages for each virus are reported without making a distinction between the states included in the study (Table 3). In general, arbovirosis significantly decreased year by year (P<0.05). Infections caused by DENV showed a drop in 2016, and it started to increase again in 2017, although without statistical significance (P>0.05).

**Table 3:** Positivity found for each virus.

	Total N = 1038 (%)	CI (%)	2015 N = 346 (%)	CI (%)	2016 N = 346 (%)	CI (%)	2017 N = 346 (%)	CI (%)
**RESULTS**								
Negative	54.4	51.4–57.4	32.4	27.5–37.3	52.3	47.0–57.6	78.6	74.3–82.9
Total positives	45.6	42.6–48.6	67.6	62.7–72.5	47.7	42.4–53.0	21.4	17.1–25.7
Positive 1 virus	43.4	40.4–46.4	62.4	57.3–67.5	46.8	41.5–52.1	20.8	16.5–25.1
Positive 2 or more viruses	2.2	1.3–3.1	5.2	2.9–7.5	0.9	-0.1–1.9	0.6	-0.2–1.4
**DENV Totals**	12.2	10.2–14.2	13.6	10.0–17.2	11.0	7.7–14.3	12.1	8.7–15.5
DENV 1	4.2	3.0–5.4	3.5	1.6–5.4	0.9	-0.1–1.9	8.4	5.5–11.3
DENV 2	4.9	3.6–6.2	6.4	3.8–9.0	5.5	3.1–7.9	2.9	1.1–4.7
DENV 3	0.7	0.2–1.2	0.0	0.0	1.7	0.3–3.1	0.3	-0.3–0.9
DENV 4	0.9	0.3–1.5	2.3	0.7–3.9	0.3	-0.3–0.9	0.0	0.0
DENV NS	1.9	1.1–2.7	2.0	0.5–3.5	2.6	0.9–4.3	1.2	0.1–2.3
**CHIKV**	20.6	18.1–23.1	59.0	53.8–64.2	2.9	1.1–4.7	0.0	0.0
**ZIKV**	14.6	12.5–16.7	0.0	0.0	34.7	29.7–39.7	9.2	6.2–12.2

N: total samples analyzed. CI: confidence interval. DENV NS: non-serotyping dengue.

Four different serotypes of DENV circulated without an apparent tendency between them. Serotype 2 was the most frequent, serotype 3 the least. In all years, variants that could not be associated with a specific serotype were detected, so they were defined as non-serotypeable DENV (DENV NS).

CHIKV positivity was higher in 2015 than in 2016 (P<0.05), and in 2017, no positive case was identified. ZIKV was not detected in 2015 and had a significantly higher positivity in 2016 than in 2017 (P<0.05).

**Fig 1:**
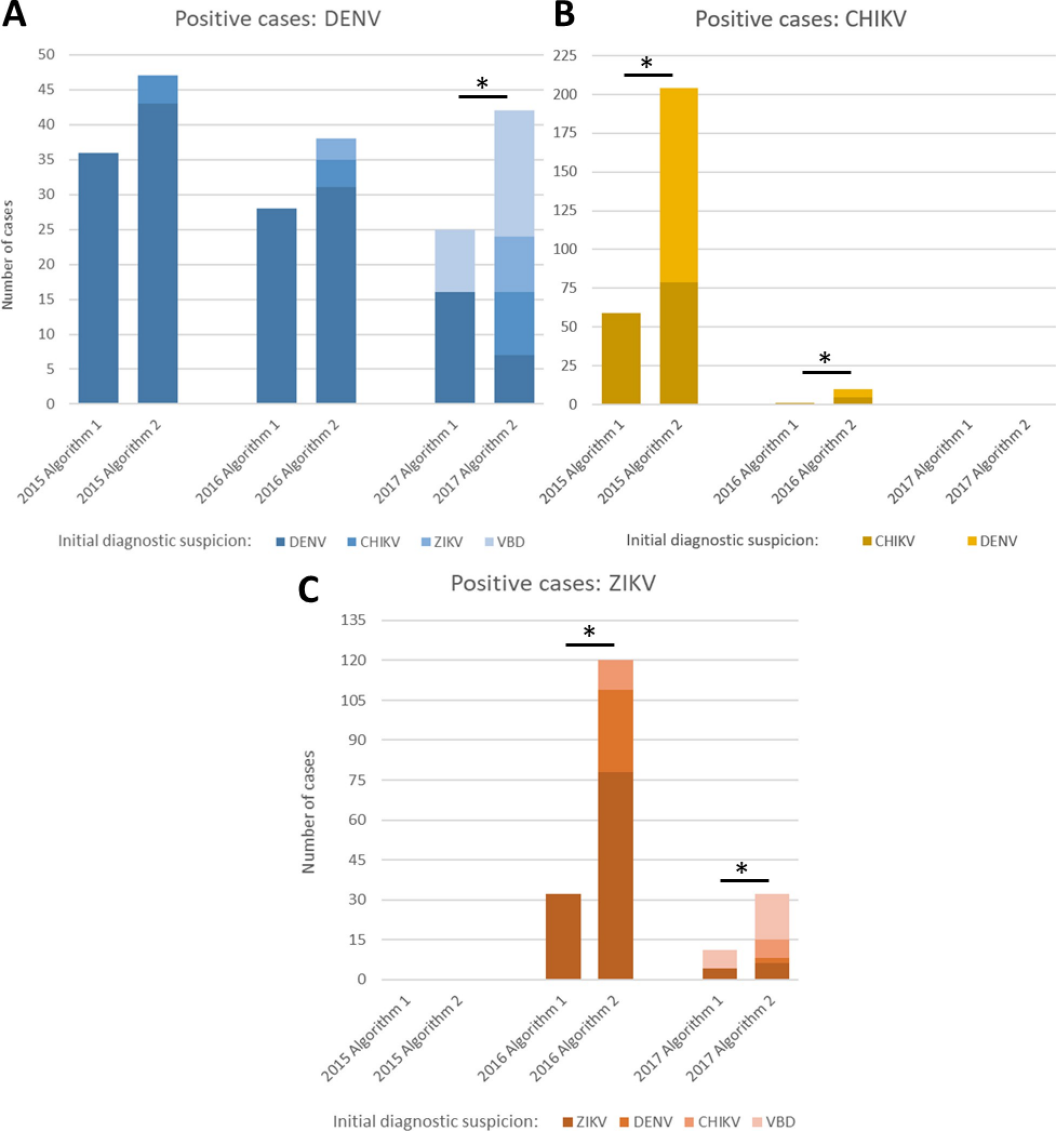
Positive cases for each Virus/Year/Algorithm. The graphs show the positivity for each virus: (A) DENV. (B) CHIKV. (C) ZIKV. **Algorithm 1:** Uniplex RT-qPCR reactions for each virus, with the restriction of performing only one reaction for each sample according to the initial diagnostic suspicion (details in S1 Fig). **Algorithm 2:** Multiplex RT-qPCR reaction that simultaneously detected the presence of the three viruses was performed on all samples, regardless of the initial diagnostic suspicion. *Significant differences (P<0.05) between the proportion of positive cases detected with algorithms 1 and 2. Note: in the last quarter of 2017, when the multiplex algorithm was implemented for all cases suspected of some vector-borne disease (VBD), a third-party kit was used for the diagnosis, and only from the second half of 2018, the LCE started using the TaqMan Zika Virus Triplex Kit also used in the present study.

With the new multiplex algorithm, 243.8% as many cases of arbovirus were identified as originally reported. The new cases identified were derived both from the lack of concordance between the initial diagnostic suspicion and the final result, as well as from a lack of concordance between the results obtained by the different algorithms. In Fig 1, we can see both the increase in overall positivity and the proportion of this due to each of the factors outlined above. With the exception of DENV in 2015 and 2016, the differences in positivity with algorithms 1 and 2 were statistically significant in all cases (P<0.05).

In the case of DENV in 2015 (Fig 1A) and ZIKV in 2016 (Fig 1C), the main difference in positivity was due to the lack of concordance between the result of both agorhythms, while the differences due to diagnostic suspicion became more relevant for DENV in 2016 and 2017, as well as for CHIKV in 2015 (Fig 1B).

### Coinfections

**Fig 2:**
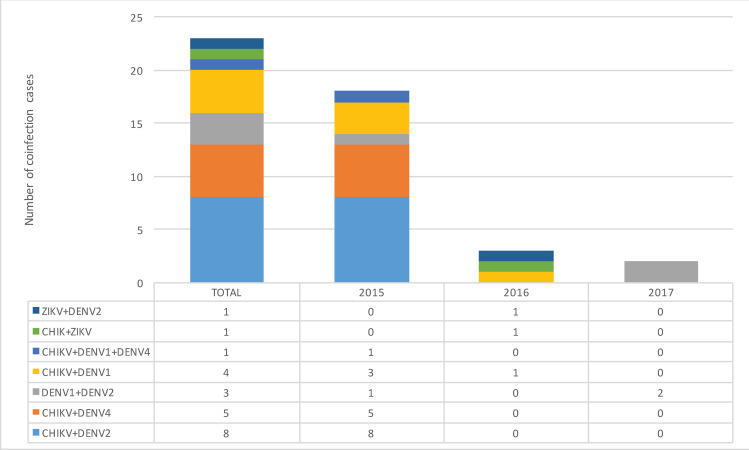
Coinfections detected in the study. The different combinations of viruses identified in cases of coinfection are broken down in the bars during the entire period of study.

In the study, 23 cases of coinfection were identified, as shown in Fig 2, equivalent to 2.2% (CI: 1.3–3.1) of the total samples analyzed and 4.9% (CI: 3.0–6.8) of the positive samples. In 78.3% (CI: 61.5–95.1) of coinfection cases, CHIKV and DENV were involved, representing 38.3% (CI: 24.4–52.2) of the cases of dengue and 8.8% (CI: 4.9–12.7) of the cases of chikungunya in 2015. In this year, 78.3% (CI: 61.5–95.1) of all coinfections were concentrated, a significantly higher percentage than that found in the following years (P<0.05), and the only coinfection caused by three viruses (CHIKV + DENV1 + DENV4) was identified.

In 2017, with the decrease in positivity for CHIKV and ZIKV, only two coinfections involving the same DENV serotypes (DENV1 + DENV2) were identified (Fig 2).

### Estimation and comparison of incidence

To determine the changes in the circulation of DENV by the arrival of CHIKV and ZIKV, since analyzing the sensitivity of the techniques was not an objective of this study, in all of the following calculations, the difference due to the sensitivity of the technique was eliminated, and only the difference derived from an erroneous diagnostic suspicion was used.

As shown in Table 4, the incidence rate of dengue in 2012–2014 was much higher than the rates estimated in any of the years analyzed, even when the multiplex algorithm was used (P<0.05). On the other hand, although without statistical significance, the dengue incidence rate rose again in 2017 (P>0.05), when no reported cases were caused by CHIKV and ZIKV incidence rate was significantly lower than in 2016.

**Table 4:** Estimation of the number of cases and the incidence rate for infection cases caused by DENV, CHIKV and ZIKV.

	Mean 2012–2014 Cases/(rate)	2015 Cases/(rate)		2016 Cases/(rate)		2017 Cases/(rate)	
	Algorithm 1	Algorithm 1	Algorithm 2	Algorithm 1	Algorithm 2	Algorithm 1	Algorithm 2
DENV	20545 (153.8)	6226 (45.6)	6745 (49.5)	4015 (29.2)	5021 (36.5)	2764 (19.9)	5527 (39.8)
CHIKV	*NA	29615 (217.2)	92359 (677.4)	2719 (19.8)	16317 (118.5)	*NA	*NA
**ZIKV**	*NA	*NA	*NA	15240 (110.7)	31899 (231.8)	2209 (15.9)	3682 (26.5)

**Algorithm 1:** Uniplex RT-qPCR for each virus, with the restriction of performing only one for each sample according to the initial diagnostic suspicion. **Algorithm 2:** Multiplex RT-qPCR that simultaneously detected the presence of the three viruses was performed on all samples, regardless of the initial diagnostic suspicion. For the 2012–2014 average, the data generated in the LCE were used as a basis. *NA: not applicable.

### Seasonality

**Fig 3:**
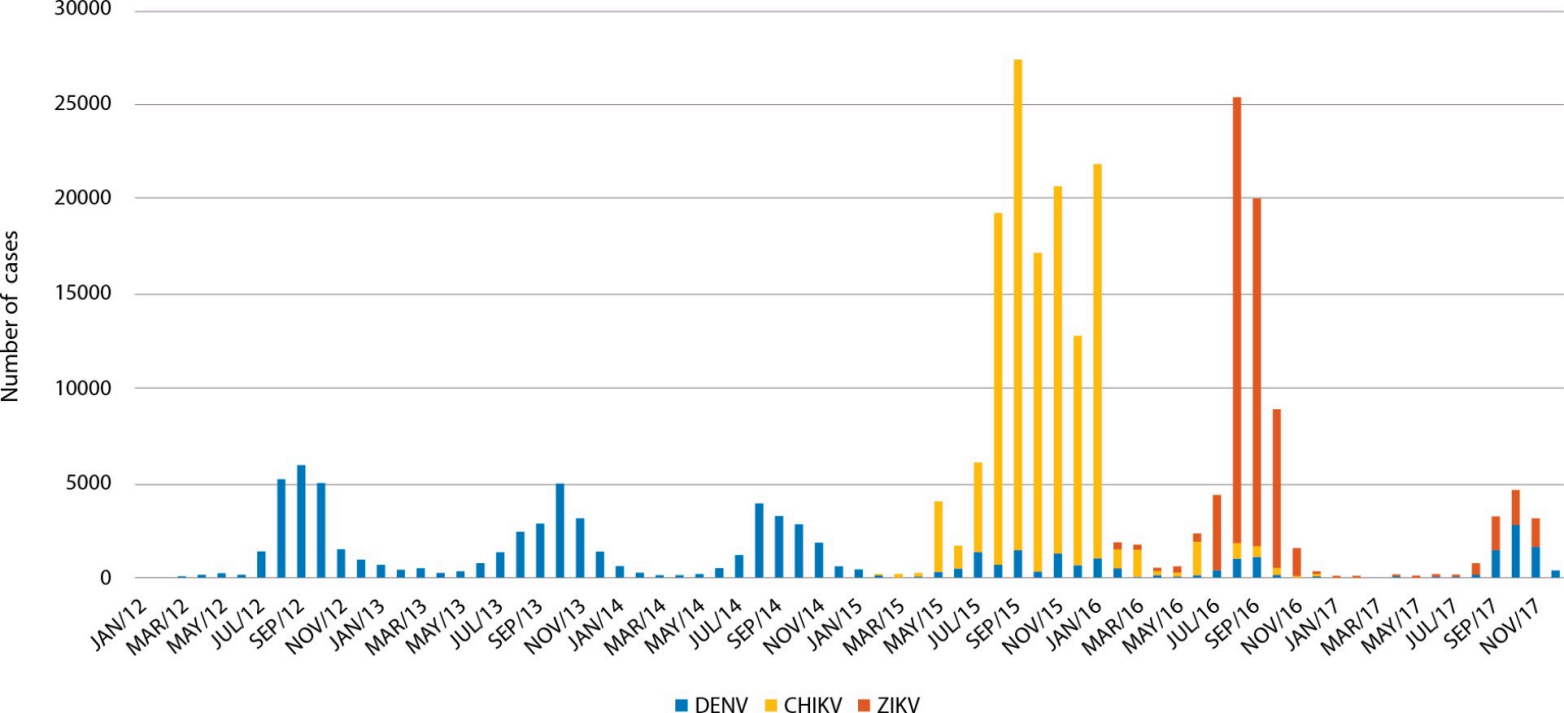
Seasonality of viruses tested. The graph was built from the estimated annual incidents generated with the data obtained in this work for each of the viruses, distributed monthly from 2012 to 2017.

When analyzing the seasonality of DENV, it seemed that this virus had been circulating with different intensities, but steadily, despite the significant decrease in incidence in 2015 and 2016 (P<0.05). CHIKV and ZIKV arose with sudden onset and high incidence rates, although for a short time, coinciding with the periods of lower DENV (Fig 3). The sum of confirmed cases of the three viruses during the period analyzed showed that arboviruses overall increased significantly (P<0.05).

**Fig 4:**
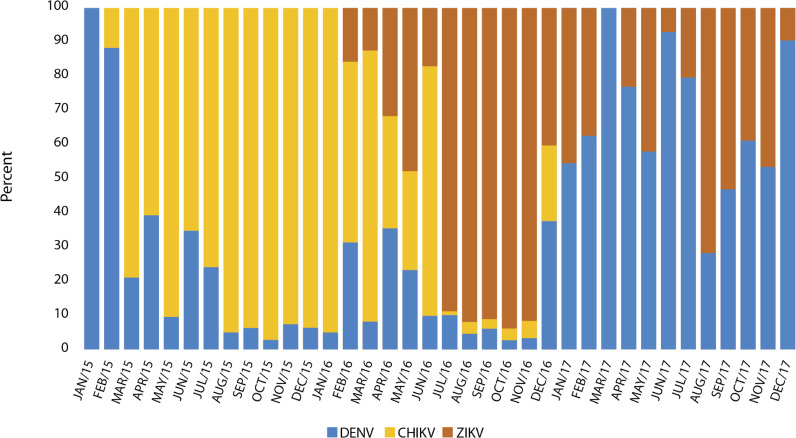
Contributions of DENV, CHIKV, and ZIKV to the etiology of arbovirosis 2015–2017. The graph was built from the estimated annual incidents generated with the data obtained in this work for each of the viruses, distributed monthly from 2015 to 2017.

When analyzing the composition of all the arboviruses together, between 2015 and 2017 (Fig 4), a marked decrease of DENV is noted with the arrival of CHIKV. Furthermore, as of February 2016, with the arrival of ZIKV, the three viruses coexisted, followed by the predominance of the latter between July and November of the same year. Finally, throughout 2017, ZIKV disappeared, while the prevalence of DENV increased.

### Endemic channel

**Fig 5:**
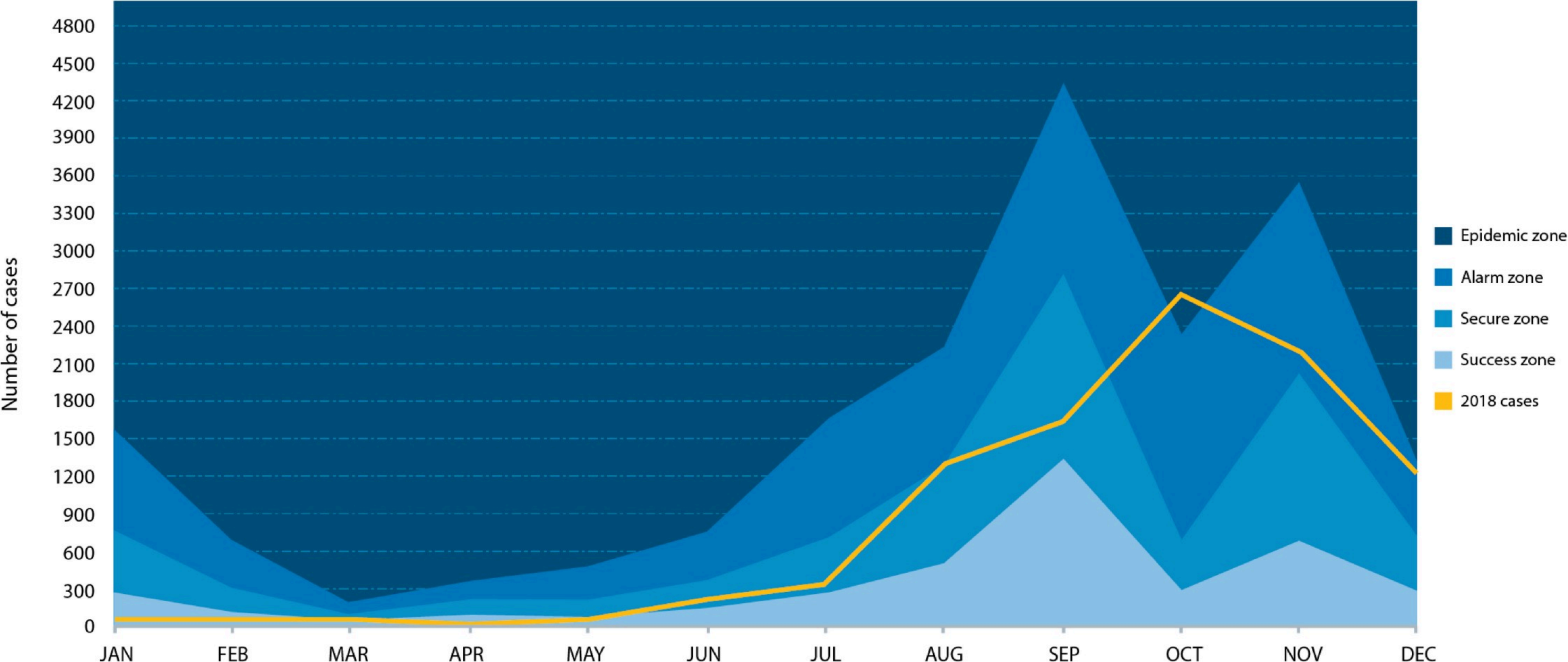
Dengue endemic channel updated for 2018. The endemic channel was built with the estimated incidences of 2015–2017, over which the estimated monthly cases for the year 2018 were plotted.

A dengue endemic channel was generated from the data estimated for 2015–2017 (Fig 5). Analyzing this channel showed that the month most affected in terms of the incidence of DENV by the introduction of CHIKV and ZIKV in these years was October. When graphing the estimated data for 2018, the line exceeded the alarm threshold in October, reaching the epidemic area, which shows a recovery in the number of dengue cases compared to the years of co-circulation with CHIKV and ZIKV (see Fig 3).

## Discussion

DENV is considered endemic to many states of Mexico, mainly in the south-southeast region of the country, and occurs more frequently in the months of July to December, when rains facilitate the proliferation of the vector [17]. However, with the introduction of CHIKV at the end of 2014 and the arrival of ZIKV at the end of 2015, significantly fewer cases of dengue were observed than in the three previous years when analyzing the results reported by the General Directorate of Epidemiology [10–15]. It is possible that the decrease in the number of reported cases of dengue is related to the similarity of the clinical picture of these infections, since the symptoms are very similar and, during years of CHIKV and ZIKV introduction, an independent diagnostic algorithm was used for each virus [6–8]; that is, the physician had to request the confirmatory diagnostic test for the initial clinical suspicion based on symptoms, and in the case of a negative result, no other test was performed for a differential diagnosis. This led to an underestimation of the incidence not only of DENV but also of CHIKV and ZIKV, not to mention the limitation of not being able to detect cases of coinfection. The diagnostic algorithm was only corrected in September 2017, and the new algorithm included the multiplex diagnosis technique to identify all three viruses in one sample [9].

This is the first study performed in Mexico that intentionally sought, without bias from underestimation of cases, to understand whether and how the arrival of CHIKV and ZIKV in the country led to the observed impact on the incidence of dengue.

When analyzing the selected samples, a total of 473 positive cases were found for at least one of the three arboviruses, representing 45.6% positivity and a significant increase of 2.4 times over the 194 cases reported as positive before the study, demonstrating the high underestimation in the incidence of arbovirosis reported between 2015 and 2017.

Even though all the RT-qPCR techniques used in this study are recommended by the Centers for Disease Control and Prevention for the confirmatory diagnosis of arbovirosis, upon reanalysis of the samples with the multiplex technique, there was disagreement in the results based on the same initial diagnostic suspicion. This is because, in some cases, an initial negative result for a given virus was positive when using the multiplex technique implemented in September 2017, although it is noteworthy that only ZIKV in 2016 showed a significant difference derived from this factor. In the absence of other studies that support this difference in the sensitivity of the techniques, and since it was not an objective of this study to evaluate this hypothesis, this difference was eliminated from the subsequent analyses.

Upon analyzing only the new positive cases derived from an inadequate diagnostic suspicion, it became evident that the distinction between the symptoms of the three infections represents a challenge for physicians when they must choose a single technique or must rely solely on clinical manifestations, since it was found that a high percentage of cases caused by CHIKV and ZIKV were sent for suspected dengue. While, when analyzing the new cases positive for DENV, we saw that, as the awareness about the circulation of the other two viruses increased, so did the number of suspected cases erroneously sent for those viruses.

For all viruses and during the three years covered by the study, new positive cases were found that could not have been detected with the previous algorithm, that is, they were derived from an erroneous diagnostic suspicion. Contrary to what was expected, the highest proportions of these cases were identified in DENV-suspect samples that were positive for CHIKV or ZIKV.

When the incidence rates were estimated, it was found that the extra positivity detected for DENV with the new multiplex algorithm did not fully explain the observed decline during the years under study. In 2017, the estimated incidence rate of dengue was significantly higher than in 2016, coinciding with the virtual disappearance of CHIKV and the rapid decline of ZIKV. Due to the characteristics of the study, it was not possible to know the cause of this phenomenon.

However, the phenomenon of the decrease in the circulation of one arbovirus with the appearance of others has also been observed in a study carried out in Recife, Brazil, in 2015–2016. In that study, 263 blood samples from patients with symptoms suggestive of an arboviral disease were analyzed to confirm infection with DENV, ZIKV, or CHIKV, and they detected the decrease in the circulation of ZIKV coinciding with the arrival of CHIKV. They also confirmed a decrease in dengue cases due to the circulation of the other two arboviruses, even though initially the official agencies had reported a strong increase in the circulation of DENV in the region. Their analysis showed that in reality they were cases of ZIKV [18].

As for the seasonality of the viruses evaluated in this study, on two occasions, a significant rise and fall in the number of total cases of arbovirosis was seen. This behavior was presented in 2015 by the introduction of CHIKV and again in 2016, this time by the arrival of ZIKV. This large variability in the number of cases, which is more than quadruple the average number of positive cases of 2012–2014, cannot only be explained by a shift in dengue cases towards one of the other arboviruses. We could hypothesize that this increase in total arbovirosis is attributed to the immunological susceptibility of the population during the first outbreak of each virus [19].

On the other hand, the virtual disappearance of CHIKV and ZIKV after the first outbreak may have been due precisely to the absence of this susceptibility. For CHIKV, for example, a study conducted by Laras et al. [20] in 2005, which sought to describe the temporal and spatial spread of CHIKV epidemic through the Indonesian archipelago, proposes that the adaptive immune response against this virus after a primary infection confers protection against reinfection [20]. In addition, the virtual disappearance of CHIKV and ZIKV could also be the result of herd immunity, where the protection of a group against an infectious disease depends on the presence of a critical mass of individuals who are immune to it.

As a simple assumption, there could be an association between the rapid decrease in zika cases and a certain degree of cross-protection derived from the antigenic similarity between this virus and the different DENV serotypes. In a country like Mexico, where marked changes in dengue seasonality was noted during the period of co-circulation with other arboviruses, serological surveys would be necessary to retrospectively understand issues related to an immunoprotective effect. With DENV, although there is already evidence of cross protection between serotypes, it is believed that this protection only lasts approximately 2 years, which may explain its fluctuating incidence but its constant presence from one year to another [21]. Similarly, more studies related to the joint interaction of arboviruses with mosquitoes and the host would also be necessary, so that the co-infections detected in this work could also be understood.

Next, the etiological composition of the arboviruses attributed to these three viruses in 2015–2017 was examined, where the impact observed in dengue could be appreciated, first due to the introduction of CHIKV in the country in 2015, and then in 2016 with the introduction of ZIKV. However, as the frequency of these two new viruses decreased, the cases of dengue began to increase, as is also seen in the endemic channel. Thus, it is important to closely monitor the behavior not only of DENV but also of CHIKV and ZIKV in upcoming years and to discuss adequate measures to avoid the increase in cases and the outbreak of new arboviruses. Another important point to consider is whether the infected population during CHIKV and ZIKV epidemics from 2015 to 2017 can suffer future immunopathological consequences, because it is not yet clear whether previous arboviral infections may represent a risk factor for atypical manifestations [22].

The recession of CHIKV and ZIKV does not mean that they cannot reemerge, although it may still be a few years before the immunity acquired by the population of the endemic areas decreases, so we expect that the number of cases of these viruses will remain low, or even null, until new susceptible individuals accumulate that could help trigger a new outbreak. For example, CHIKV has presented epidemic peaks in seven- to eight-year cycles, with reports of silencing of up to three decades in countries such as Uganda [20].

With respect to coinfections, there are several theories about how they can occur [23], in fact, some studies have already tried to reproduce coinfections in mosquitoes and have shown that there is no strong interference in the replication of viruses [24–26]. The arrival of CHIKV and ZIKV in Mexico has demonstrated the increasing possibility of joint transmission of the different arboviruses, making differential diagnosis even more urgent, despite the fact that in our study the number of coinfections detected was not high.

Finally, it would also be important to identify the reason for the existence of DENV variants that could not be serotyped. Our study demonstrates the need to design new primers and probes for better detection and serotyping of DENV, as well as to monitor the evolution of this virus and the appearance of important mutations.

## Conclusions

This study determined for the first time the degree of underestimation of arboviruses in Mexico and the degree of observed impact on the incidence of dengue after the introduction of two new arbovirus in the country. In addition, for the first time, co-infections with these viruses have been reported, which unfortunately the clinical importance is not yet known. With all of the above, the information generated in this study enriches the country with more precise epidemiological information on these arboviruses, which can help improve estimates of burden, morbidity and resource allocation, as well as providing information to design better diagnostic algorithms, given the possible scenario of new outbreaks or epidemics caused by these or other arboviruses that are currently circulating in the Americas.

## Supporting information

S1 Fig Flow diagram of algorithm 1 and 2 methodology. (TIF)

S2 Fig Methodology used to calculate the incidence estimate for each virus / year. The process that was carried out was divided into 6 steps: STEP 1: obtain the value "C", which is the ratio of the proportion between the positivity before (A) and after (B) the study in the samples analyzed. STEP 2: multiply that proportion "C" by the total number of positive cases that had been reported by the LCE (D), thus obtaining the estimate of the incidence in the samples that arrived at this laboratory (E). However, only a percentage of suspected cases are sent to the LCE for diagnostic confirmation (F), so it was necessary to make the calculation shown in STEP 3 to obtain the estimate for the incidence at the IMSS (G). STEP 4: obtain "I", which is the ratio of the proportion between the estimate (G) and the total number of IMSS beneficiaries (H). STEP 5: multiply the proportion "I" by the total number of the state’s inhabitants "J", thus finding the estimate of the number of cases for the 3 states analyzed (together) in this work (K). To report the incidence value per 100,000 inhabitants, better known as the incidence rate (L), the calculation shown in STEP 6 was carried out. The number of beneficiaries used in 2012 was 4633935, in 2013 it was 4777388, in 2014 it was 4548136, in 2015 it was 5123954, in 2016 it was 4605524 and in 2017 it was 4841681. For step 5 the population estimates published by the National Population Council (CONAPO) were used. For (F), the sampling percentage within the IMSS is 30% for the DENV and 10% for CHIKV and ZIKV.
(TIF)

S1 Table Primers and probes used for DENV serotyping. (DOCX)

S1 Data Raw data. (XLSX)
