# Supplementary figures and images for: Impact of the introduction of chikungunya and zika viruses on the incidence of dengue in endemic zones of Mexico
Source: PLoS Negl Trop Dis. 2021 Dec 2;15(12):e0009922. doi: 10.1371/journal.pntd.0009922 (PMC8638990; doi:10.1371/journal.pntd.0009922)

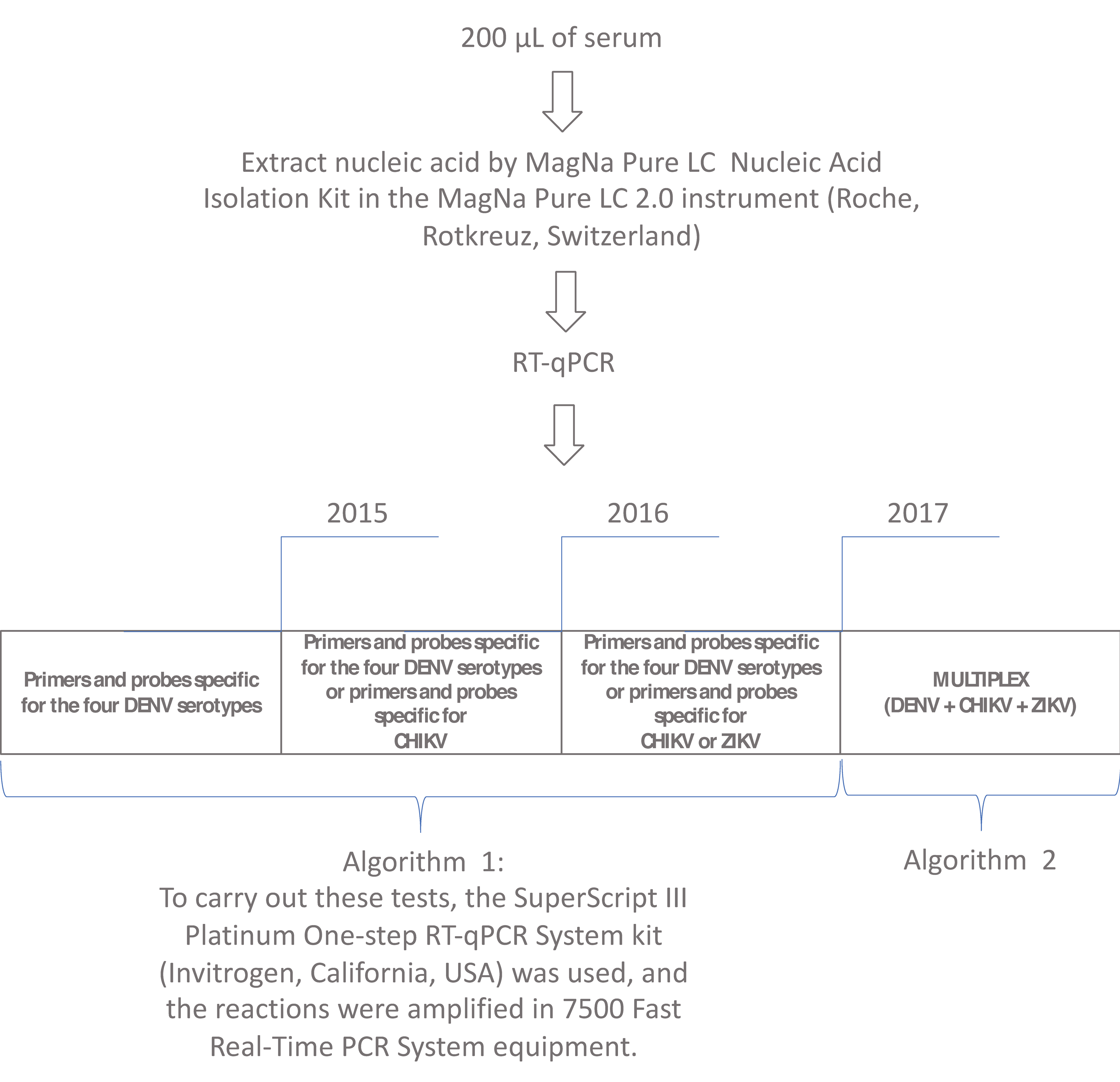

Supplement: S1 Fig — (TIF) [file pntd.0009922.s001.tif]

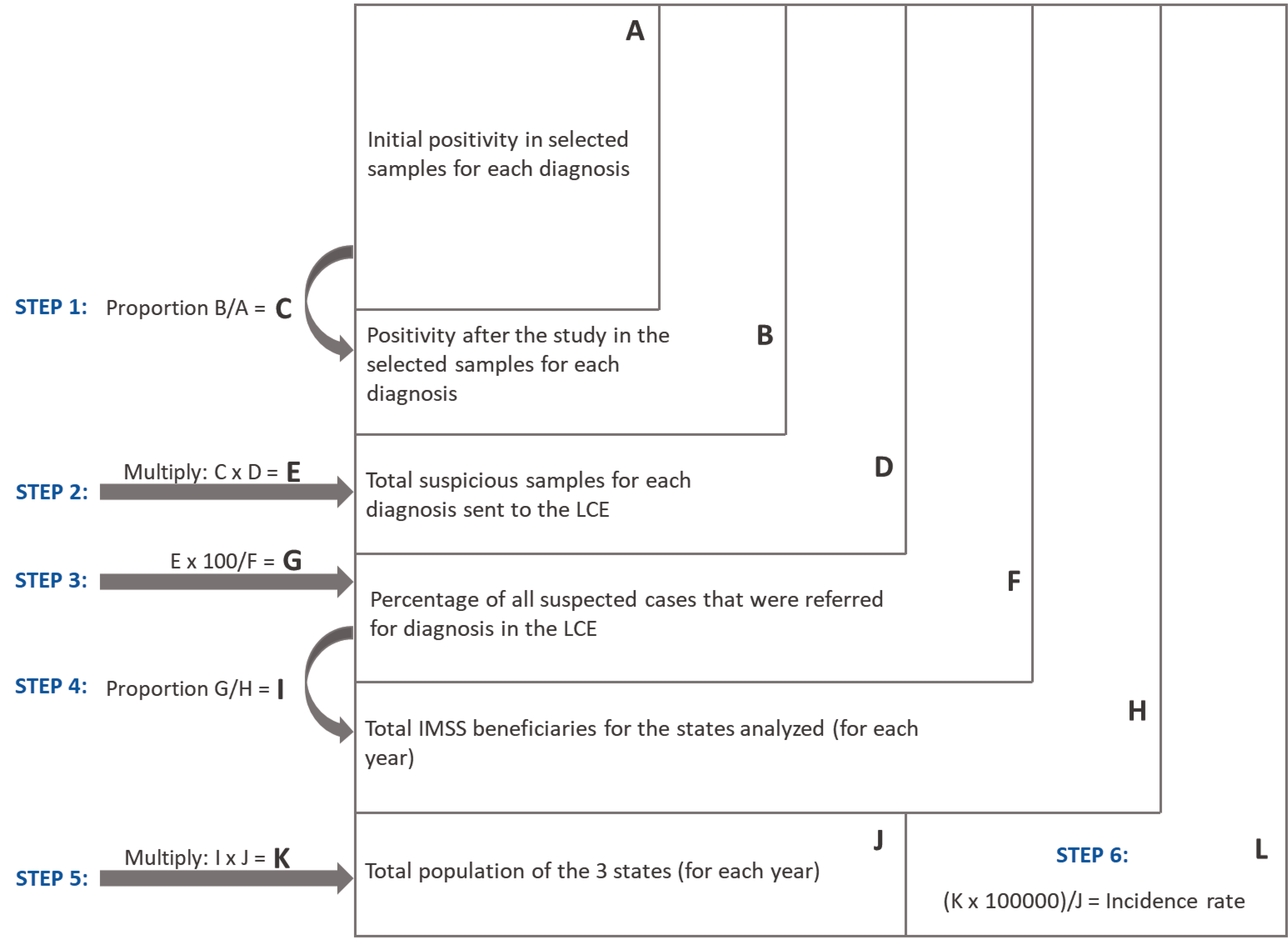

Supplement: S2 Fig — The process that was carried out was divided into 6 steps: STEP 1: obtain the value "C", which is the ratio of the proportion between the positivity before (A) and after (B) the study in the samples analyzed. STEP 2: multiply that proportion "C" by the total number of positive cases that had been reported by the LCE (D), thus obtaining the estimate of the incidence in the samples that arrived at this laboratory (E). However, only a percentage of suspected cases are sent to the LCE for diagnostic confirmation (F), so it was necessary to make the calculation shown in STEP 3 to obtain the estimate for the incidence at the IMSS (G). STEP 4: obtain "I", which is the ratio of the proportion between the estimate (G) and the total number of IMSS beneficiaries (H). STEP 5: multiply the proportion "I" by the total number of the state’s inhabitants "J", thus finding the estimate of the number of cases for the 3 states analyzed (together) in this work (K). To report the incidence value per 100,000 inhabitants, better known as the incidence rate (L), the calculation shown in STEP 6 was carried out. The number of beneficiaries used in 2012 was 4633935, in 2013 it was 4777388, in 2014 it was 4548136, in 2015 it was 5123954, in 2016 it was 4605524 and in 2017 it was 4841681. For step 5 the population estimates published by the National Population Council (CONAPO) were used. For (F), the sampling percentage within the IMSS is 30% for the DENV and 10% for CHIKV and ZIKV. (TIF) [file pntd.0009922.s002.tif]
