# Supplementary material for: Impact of the introduction of chikungunya and zika viruses on the incidence of dengue in endemic zones of Mexico
Source: PLoS Negl Trop Dis. 2021 Dec 2;15(12):e0009922. doi: 10.1371/journal.pntd.0009922 (PMC8638990; doi:10.1371/journal.pntd.0009922)
Supplement: S1 Table — (DOCX) [file pntd.0009922.s003.docx]

**Supplementary Table 1**. Primers and probes used for DENV serotyping.

| **SEROTYPE** | **PRIMERS / PROBES** | **SEQUENCE (5′-3′)** | **CONC (µM)** |
| --- | --- | --- | --- |
| **DENV 1** | Forward | CAA AAG GAA GTC GYG CAA TA | 100 |
| **DENV 1** | Reverse | CTG AGT GAA TTC TCT CTG CTR AAC | 100 |
| **DENV 1** | Probe | FAM CAT GTG GYT GGG AGC RCG C BHQ-1 | 100 |
| **DENV 2** | Forward | CAG GCT ATG GCA CYG TCA CGA T | 100 |
| **DENV 2** | Reverse | CCA TYT GCA RCA CCA TCT C | 100 |
| **DENV 2** | Probe | HEX CTC YCC RAG AAC GGG CCT CGA CTT CAA BHQ-1 | 100 |
| **DENV 3** | Forward | GGA CTR GAC ACA CGC ACC CA | 100 |
| **DENV 3** | Reverse | CAT GTC TCT ACC TTC TCG ACT TGY CT | 100 |
| **DENV 3** | Probe | TEXAS RED ACC TGG ATG TCG GCT GAA GGA GCT TG BHQ-2 | 100 |
| **DENV 4** | Forward | TTG TCC TAA TGA TGC TRG TCG | 100 |
| **DENV 4** | Reverse | TCC ACC YGA GAC TCC TTC CA | 100 |
| **DENV 4** | Probe | Cy5 TYC CTA CYC CTA CGC ATC GCA TTC CG BHQ-3 | 100 |
| **RP** | Forward | CCA AGT GTG AGG GCT GAA AAG | 100 |
| **RP** | Reverse | TGT GGC TGA ACT ATA AAA GG | 100 |
| **RP** | Probe | FAM CCC CAG TCT CTG TCA GCA CTC CCT TC BHQ-1 | 100 |

CONC: concentration; RP: RNAsa P.
